# The costs and benefits of decentralization and centralization of ant colonies

**DOI:** 10.1093/beheco/arz138

**Published:** 2019-08-14

**Authors:** Dominic D R Burns, Jon W Pitchford, Catherine L Parr, Daniel W Franks, Elva J H Robinson

**Affiliations:** 1 Department of Biology, Wentworth Way, University of York, York, UK; 2 York Cross-disciplinary Centre for Systems Analysis, University of York, York, UK; 3 Department of Mathematics, University of York, York, UK; 4 Department of Earth, Ocean and Ecological Sciences, University of Liverpool, Jane Herdman Building, Liverpool, UK; 5 Centre for African Ecology, School of Animal, Plant and Environmental Sciences, University of the Witwatersrand, Wits, South Africa; 6 Department of Zoology and Entomology, University of Pretoria, Hatfield, Pretoria; 7 Department of Computer Science, Deramore Lane, University of York, York, UK

**Keywords:** collective decision-making, decentralization, dynamic networks, polydomy, social insects, social networks

## Abstract

A challenge faced by individuals and groups of many species is determining how resources and activities should be spatially distributed: centralized or decentralized. This distribution problem is hard to understand due to the many costs and benefits of each strategy in different settings. Ant colonies are faced by this problem and demonstrate two solutions: 1) centralizing resources in a single nest (monodomy) and 2) decentralizing by spreading resources across many nests (polydomy). Despite the possibilities for using this system to study the centralization/decentralization problem, the trade-offs associated with using either polydomy or monodomy are poorly understood due to a lack of empirical data and cohesive theory. Here, we present a dynamic network model of a population of ant nests which is based on observations of a facultatively polydomous ant species (*Formica lugubris*). We use the model to test several key hypotheses for costs and benefits of polydomy and monodomy and show that decentralization is advantageous when resource acquisition costs are high, nest size is limited, resources are clustered, and there is a risk of nest destruction, but centralization prevails when resource availability fluctuates and nest size is limited. Our model explains the phylogenetic and ecological diversity of polydomous ants, demonstrates several trade-offs of decentralization and centralization, and provides testable predictions for empirical work on ants and in other systems.

## Teaser

Centralization and decentralization of resources in ant colonies have several costs and benefits. We use a dynamic network model of a population of ant nests to demonstrate that there are several important costs and benefits of centralization and decentralization, which explains the phylogenetic and ecological diversity of methods for distribution of resources across ant species.

## INTRODUCTION

Centralization and decentralization are opposing strategies for the spatial organization of resources or workers. In centralized systems, the resources are located in a single site, while in decentralized systems, resources are dispersed across multiple sites. Decentralization and centralization have many interacting costs and benefits, making it difficult to identify which should be adopted in a given context (Robinson 2014; Bernstein and Turban 2018; Ireland and Garnier 2018). One potential source of insights for the trade-offs between centralization and decentralization are ant colonies (Hölldobler and Lumsden 1980; Ireland and Garnier 2018).

**Figure 1:**
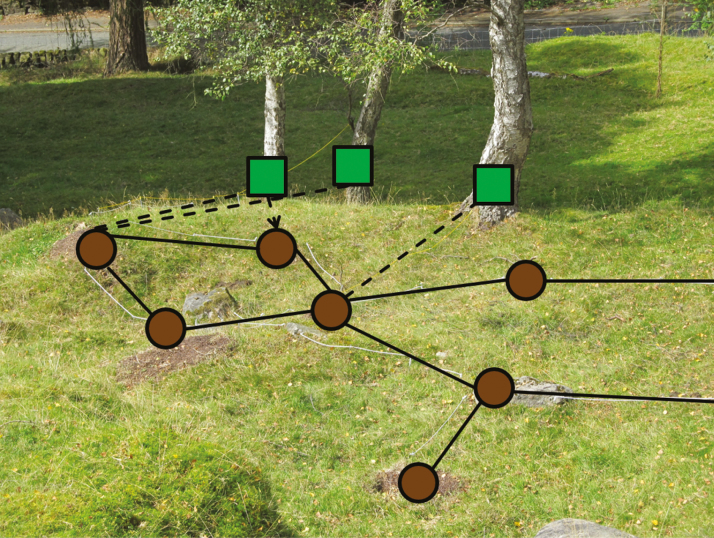
A polydomous colony of wood ants foraging on aphids that live in trees. For clarity, nests are marked with brown circles, food sources (trees) are marked with green squares, foraging trails are marked with dashed lines, and inter-nest trails are marked with solid lines.

The workforce and resources of most ant colonies are centralized in a single nest, which is known as monodomy. However, some ant species decentralize their colonies by dividing into several semi-autonomous subgroups that inhabit multiple nests, known as polydomy (Debout et al. 2007) (Figure 1). Decentralization through polydomy is hypothesized to confer several benefits on colonies including: 1) reducing the risk of colony extinction, through spreading risk (Le Breton et al. 2007; Van Wilgenburg and Elgar 2007; Robinson 2014); 2) enabling colonies to overcome population limits imposed by structural or organizational limitations on nest size (Van Wilgenburg and Elgar 2007; Robinson 2014); 3) improves colony foraging and defense through nests being well-distributed through the foraging area (Hölldobler and Lumsden 1980; Davidson 1997; Holway and Case 2001; Schmolke 2009; Lanan et al. 2011; Cook et al. 2013; Stroeymeyt et al. 2017); 4) buffering the effects of local environmental variability (Schmolke 2009; Cook et al. 2013; Robinson 2014); and 5) enabling colonies to benefit from a large colony size, without the associated reductions in productivity (Kramer et al. 2014; Stroeymeyt et al. 2017). Despite these benefits, most ant colonies are monodomous. The reason for the relative abundance of monodomy may be due to some key costs of spreading across multiple nests, including: 1) reduced defensive abilities, as defenders may be spread too thinly (Robinson 2014); 2) impaired information transfer between individuals when they are spread across multiple nests (Cook et al. 2013); and 3) costs of moving resources between nests due to predation or resource theft (Snyder and Herbers 1991; Robinson 2014).

The many inter-related costs and benefits of decentralization of an ant colony’s workforce make understanding the reasons that various ant species have evolved polydomy challenging to test in the field or laboratory. Furthermore, the species of ants that demonstrate polydomy are phylogenetically and ecologically diverse, as polydomy has evolved multiple times, seemingly in response to different selective pressures (Debout et al. 2007). Consequently, no single hypothesis for the adaptive benefits of polydomy fits with the evidence from every case of polydomy (Robinson 2014). One key difficulty is that we lack clear predictions of how colony spatial structure should respond to different environmental pressures. One method that has been used, with some success, is mathematical models which compare the success of polydomy and monodomy in different situations; however, current models of nesting organization have been designed to test a single hypothesis and cannot be generalized to most cases of polydomy (Höfener et al. 1996; Schmolke 2009; Cook et al. 2013; Bottinelli et al. 2015). Consequently, models that provide proof-of-concept tests and verifiable predictions for experimental and observational research are a necessary and relatively unexplored method for research into the trade-offs between centralization and decentralization of ant nests.

Here, using a dynamic network model, we take a novel approach which investigates multiple hypotheses for the ecological benefits of spatial decentralization of ant colonies.

## METHODS

### Model overview

**Figure 2:**
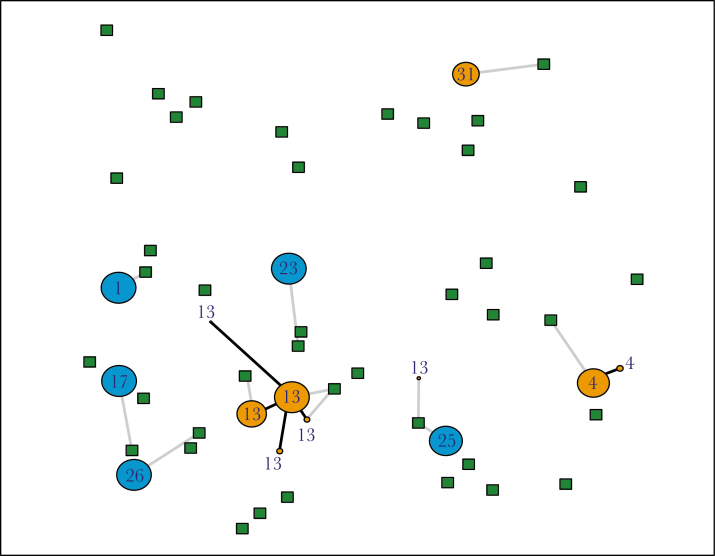
Graphical representation of the model. Circles represent nests and squares represent food sources. Nest color indicates whether the nest belongs to a polydomous (orange) or a monodomous (blue) colony, and nest number is the colony identity of that nest. Gray lines indicate foraging connections and black lines indicate inter-nest connections.

We model the dynamics of a population of ant colonies, some of which have a polydomous colony organization and others a monodomous colony organization. The model considers ant colonies as networks with nodes representing nests and food sources, and connections representing resource exchange between those nodes (e.g., Figure 2). Over time, the strategies compete against each other (see Supplementary Video for example of model running), allowing us to test various hypotheses for ecological benefits of decentralization and centralization in ant colonies by manipulating parameters of the model. As the model is stochastic, we run multiple replicates under each set of conditions; every replicate is a unique realization of the model. We use this model to formalize five existing hypotheses (Table 1) for the adaptive benefits of polydomy and identify ecological conditions where each is likely to be important in driving the evolution of polydomy.

**Table 1:** Hypotheses and factors changed in the model to test logic of these hypotheses

Hypothesis	Change in model	Levels
Polydomy is favored when:		
(1) the costs of foraging are high	Foraging cost	Low, high
(2) nests are limited in size	Nest-level carrying capacity	Low, high
(3) food sources are clustered	Food source distribution	Clustered, random
(4) there is a risk of stochastic nest destruction	Probability of stochastic nest destruction per season	None, 1%
(5) food sources vary in availability	Food source productivity	Constant, fluctuating

Here, we present an overview of how the model works; a fully detailed model description can be found in Supplementary Appendix 1. The model processes are based on empirical observations of the facultatively polydomous wood ant *Formica lugubris* (Ellis and Robinson 2014). Empirically measured values for several of the model parameters are unavailable, but the model results are not highly sensitive to these parameters (Supplementary Appendix 9). The model is implemented in R version 3.4.1 (R Core Team 2017). Although implemented to investigate polydomous wood ants, our general modeling framework describes interactions between dynamically varying networks (nests and food sources in our case) and is readily adapted; the modular nature of the processes involved (as detailed below) facilitates such adaptation.

### Environment and agents

In the model, there are nests and food sources, each of which has a fixed location (e.g., Figure 2). At the start of each replicate of the model, nests are randomly distributed in space, while food sources can be either randomly distributed or clustered depending on the condition (see Table 1—Hypotheses and Supplementary Appendix 2). Nests contain resources that are implicitly assumed to be ants capable of foraging. Each nest also has a colony organization, which can be either polydomous or monodomous. At the start of each replicate, half of the nests are polydomous and half are monodomous. Food sources have a fixed location throughout each replicate of the model. Food sources produce food at either a constant rate, or at a rate that fluctuates over time (Table 1).

### Foraging connections

Nests make foraging connections to food sources. These are representative of foraging trails commonly found in wood ants (Ellis and Robinson 2014). Nests use food received from foraging connections to increase the quantity of resources (equal to ants) in the nest. However, not all foraging connections are profitable. The profitability of a connection is determined by the availability of resources at the food source, the number of ants foraging on it, and its length, with longer connections being costlier. Such costs may include factors such as energy used by foragers, maintenance costs, and time costs.

### Inter-nest connections

Nests that belong to the same polydomous colony can form connections to each other, allowing them to take food. As with foraging connections, each connection can be costly if it is long or if there are few resources available.

### Competition

If two nests from different colonies make a connection to the same food source, they compete through interference competition for the food. A sensitivity analysis of the effect of competition on the model results is detailed in Supplementary Appendix 3.

### Nest foundation, growth, and death

Nests are capable of “parenting” new nests using their resources. If a new nest is created by a nest with a polydomous colony organization, then it will belong to the same colony as the “parent” nest and have a connection allowing it to take resources from the “parent” nest. In contrast, nests parented by nests with a monodomous colony organization will become a new, independent colony. We assume that cooperative nests can occasionally found non-cooperative nests and vice versa. This prevents either strategy from becoming extinct. Polydomy appears to be a fairly flexible strategy in many ant species, which are facultatively polydomous (e.g., Ellis and Robinson 2014). Consequently, cooperative strategies among groups may often arise in response to local environmental conditions, rather than being inflexible.

Nests grow in size at a rate that depends on the quantity of resources that they receive from their connections. They also suffer a constant death rate and are limited in size by a “nest-level carrying capacity” (Table 1).

Nests can “die” if the nest population (resources contained in the nest) reaches below a certain threshold. Nests can also “die” randomly, which is included to represent processes such as predation, parasitism, or other stochastic causes of nest “death” (Table 1).

### Timescales

The model cycles over a set number of seasons (Supplementary Table A1.1). Seasons in our model are simply used to index time and, as such, there is no variation in conditions between seasons. In each seasonal cycle, nests grow at a rate determined by the connections that they have to food sources and other nests. At the end of each season, nests can change their connections, depending on profitability, and produce new nests. We run the simulations for multiple independent replicates, each represented by a single complete run of the model for a fixed number of seasons. A video showing a graphical representation of the model running can be found at Supplementary Video 1.

### Experimental design and hypotheses

At the end of every season, the locations, sizes, foraging connections, and inter-nest connections of every nest that is currently active are recorded. We use these data to test five different hypotheses (Table 1) by changing factors in the model in a full factorial design (i.e., all two-, three-, four-, and five-way interactions between factors are also tested). We ran 30 replicates of each unique condition (30 replicates × 32 conditions = 960 total replicates). The hypotheses and changes to the model are detailed in Table 1.

### Statistical analyses

We used a general linear model (GLM) with a binomial error distribution and a logit link as a framework to quantify how each of the factors that we change in the model affects the proportion of ants that belong to polydomous nests. Details of model selection are described in Supplementary Appendix 5. We do not report *P* values as they do not represent “statistical significance” (James et al. 2013; Wasserstein and Lazar 2016). Rather, for the fixed number of replicates under consideration, the odds ratio and confidence intervals are used to indicate factors with a clear effect. We calculate odds ratios for each of the covariates of the model to show the effect of changing them on the frequency of polydomy. The odds ratio values for each effect indicate the increase in probability that a randomly selected ant in a replicate under a certain condition belongs to a polydomous nest by changing the factor of interest (e.g., an odds ratio of 4 indicates that there is four times the probability that a randomly selected ant is from a polydomous nest when compared to replicates in which the factor of interest is set to the alternative value). As such, the odds ratio values indicate whether differences between conditions are expected to be biologically meaningful but should not be used to compare relative importance of each effect as an adaptive benefit of polydomy or monodomy, because factor levels (Table 1) cannot be standardized across factors.

## RESULTS

Of the 960 model replicates, the population survived until the final season in 91.5% of replicates. Replicates in which the population died before the final season were excluded from further analyses. Population survival under different conditions is shown in Supplementary Figure A6.1. We present multiple sensitivity analyses for model parameters in Supplementary Appendices 3 and 9.

### Foraging costs

**Figure 3:**
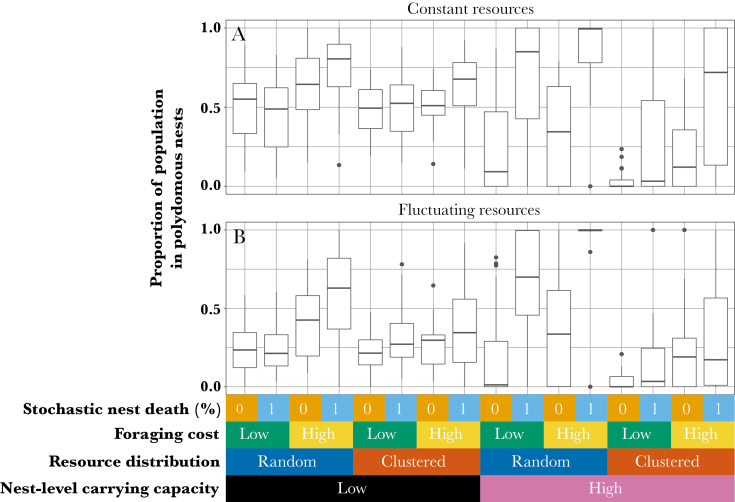
The proportion of the population in polydomous nests at the end of 500 seasons in each condition when food sources are either constant (A) or fluctuating (B) in availability. Middle lines represent median values, lower and upper hinges represent 25th and 75th percentiles, respectively, and whiskers reach to the lowest (lower whisker) or highest (higher whisker) value, with a maximum reach of 1.5 × IQR from the hinge. Values outside of this range are plotted as outliers.

Our results support the hypothesis that polydomy is favored when foraging is costly. There is a clear effect of foraging cost on the frequency of polydomy (Table 2), with polydomy being more frequent when foraging costs are high, compared with when they are low (Figure 3; effect sizes are detailed in Table 2). There are no important interaction terms between foraging cost and any of the other factors in the model.

**Table 2:** Factors included in the final GLM

Factor	*z*	Odds ratio	2.5% CI	97.5% CI
Intercept	−9.08	N/A	N/A	N/A
**Foraging trail cost (high)**	**4.99**	**2.15**	**1.59**	**2.91**
**Nest-level carrying capacity (low)**	**3.87**	**3.75**	**1.94**	**7.43**
**Food source distribution (clustered)**	**7.20**	**5.78**	**3.61**	**9.41**
**Stochastic nest destruction (high)**	**5.20**	**4.61**	**2.62**	**8.31**
Food source stochasticity (constant)	1.12	1.39	0.78	2.48
**Food source stochasticity (constant) and nest-level carrying capacity (low)**	**2.43**	**2.11**	**1.16**	**3.87**
Food source stochasticity (constant) and food source distribution (clustered)	−0.66	0.82	0.45	1.48
**Nest-level carrying capacity (low) and stochastic nest destruction (high)**	−**4.68**	**0.23**	**0.13**	**0.43**
**Nest-level carrying capacity (low) and food source distribution (clustered)**	−**3.71**	**0.31**	**0.17**	**0.58**

Odds ratio indicates the increase in probability of randomly selecting an ant from a polydomous nest when the factor (or factors) is set to the value given in parentheses in Factor; 2.5% and 97.5% CIs indicate 95% confidence intervals for each effect size. Factors with odds ratio (CIs) that do not overlap 1.00 (i.e., no effect) are highlighted in bold.

### Nest size limitations

Our results support the hypothesis that polydomy is favored when nest populations are limited in size. There is a clear effect of nest-level carrying capacity on the frequency of polydomy (Table 2), with polydomy being found more frequently when nest-level carrying capacity is low, compared to when it is high (Figure 3). We also find two-way interactions between nest-level carrying capacity and stochastic nest destruction, food source distribution, and food source stochasticity on the frequency of polydomy (details are in Table 2; descriptions of each interaction are given in the sections of “Food source distribution,” “Stochastic nest destruction,” and “Fluctuating food source availability”).

### Food source distribution

Our results support the hypothesis that polydomy is favored when food sources are clustered. In the final GLM, we find an effect of food source distribution on the frequency of polydomy (Table 2), with clustered food sources resulting in higher frequencies of polydomy than when food sources are randomly distributed (Figure 3). However, there is also an interaction between food source distribution and nest-level carrying capacity (Table 2), with clustered food sources promoting polydomy less strongly when nest-level carrying capacity is low than when nest-level carrying capacity is high.

### Stochastic nest destruction

Our data support the hypothesis that polydomy is favored when nests are at risk of stochastic nest destruction. In the final model, the frequency of polydomy is affected by stochastic nest destruction (Table 2), with stochastic nest destruction resulting in higher frequencies of polydomy (Figure 3). However, there is also an interaction between stochastic nest destruction and nest-level carrying capacity (Table 2): the increase in polydomy caused by stochastic nest destruction is smaller when nest-level carrying capacity is low than when nest-level carrying capacity is high.

### Fluctuating food source availability

Our model results support the hypothesis that polydomy is influenced by fluctuations in food source availability, but only when nest-level carrying capacity is low (Table 2). However, the direction of the effect is opposite to that hypothesized, with fluctuating food sources resulting in a lower frequency of polydomy (Figure 3). The overall effect (without interactions) of fluctuations in food source availability is not found to be important.

## DISCUSSION

Our results show that polydomous colonies perform better when resource acquisition costs are high, nest size is limited, resources are clustered, and there is a risk of nest destruction, but monodomy performs better when resource availability fluctuates temporally. Taken together, these results explain why species of ants that have polydomous colonies are phylogenetically and ecologically diverse.

Our model supports the hypothesis that decentralizing across multiple nests may be favored when acquisition of resources from the environment is costlier than resource sharing, and there is a benefit to groups that donate resources to others, for example through inclusive fitness or reciprocation. Foraging costs in our model represent energetic costs, time costs, and forager loss through predation costs. Polydomy appears to allow colonies to reduce these costs, for example by limiting the time it takes for each individual to travel to a food source. Reducing foraging costs is likely to be important in many species, including the polydomous desert ant *Cataglyphis iberica*, whose workers forage in the middle of the day when temperatures are highest and longer foraging trips may result in death (Cerda et al. 2002).

Another important determinant of the cost of foraging is recruitment, which allows colonies to quickly exploit food sources that are far from the nest without each forager having to find the food source independently. When recruitment is possible and food sources are large, then being spread across multiple nests may actually be costly, because mobilization of enough workers to exploit a food source may be not be possible from small nests (Cook et al. 2013). Here, we model the behavior of the population of individual nests, rather than individual ants. Consequently, we do not model recruitment explicitly, but instead assume that ants are able to effectively recruit to form trails to food sources.

Nest size limitation is one of the clearest causes for a colony spreading across multiple nests and is supported by the model. It is most apparent in cavity-dwelling species, and is commonly associated with seasonal polydomy, when colonies temporarily outgrow their nest (Cao 2013). Although the pressure of nest size limitations on colony size is most clear in cavity-dwelling ants, similar processes may be at work in species with high nest-size-dependent mortality or organizational constraints on nest size (Le Breton et al. 2007; Van Wilgenburg and Elgar 2007; Kramer et al. 2014).

The effect of resource distribution on success of polydomy in our model may be because polydomous colonies can monopolize clusters of permanent food sources and become difficult to displace. In contrast, monodomous colonies may be unable to monopolize large clusters of food sources due to the foraging range of individual nests being limited and may be easily invaded. Monopolization of food sources and subsequent absence of interference competition appears to be an important factor in the success of many invasive species, such as the Argentine ant (Holway and Case 2001), and of species that forage on large stable food sources, such as ant species that have mutualistic relationships with trophobionts (Ellis and Robinson 2014; Lanan 2014). This interpretation is also supported, as when competition is removed from our model, the frequency of polydomy decreases (Supplementary Appendix 3). The effect of food source distribution on the frequency of polydomy found by our model may be because when nest size is limited, being close to several food sources is less beneficial, as nests are restricted in the number of foraging and inter-nest trails they can form. Consequently, food source distribution may be less influential in determining social organization strategy when nests are only able to forage on a few food sources, or in a small area.

Our findings contrast with Cook et al. (2013) who found that monodomy performs better than polydomy when food sources are clustered. We suggest this is because Cook et al. model a single colony and do not consider inter-colony competition. In our model, polydomous colonies are often able to be more numerically dominant than monodomous colonies as they can grow larger. Consequently, when food sources are clustered, polydomous colonies in our model can monopolize clusters more easily making it difficult for competitors to invade, which may be an important mechanism in ant species that forage on large, consistent food sources, such as populations of aphids (Ellis and Robinson 2014). When interference competition is removed from our model then, like Cook et al., we find that polydomy is less prevalent when food is clustered (Supplementary Appendix 3).

Our model supports the hypothesis that polydomy allows colonies to spread the risk of nest destruction. Decentralization may allow the wider group to spread the risk of damage from external processes across multiple nests: if one nest is destroyed, the colony can still persist, provided there are multiple queens in the colony (Robinson 2014; Van Wilgenburg and Elgar 2007). Pressure from stochastic nest destruction is likely to be important in species where nests are targeted by predators (Le Breton et al. 2007; Van Wilgenburg and Elgar 2007), but could also represent other processes through which random nest destruction occurs, for example social parasitism, which may be important in many ant species (Czechowski and Godzińska 2015), or environmental damage. Some of the effect of stochastic nest destruction on the success of polydomy is lost when nest size is very limited. When nests are limited to being small, there are likely to be more of them, because individual nests use less food than when nests can grow very large, meaning that the maximum number of nests that an environment can support is higher. When this is the case, stochastic destruction of a single nest is likely to be a less detrimental event for a polydomous colony.

In our model, stochastic nest destruction is a completely random process that occurs independently for each nest. However, ecological and environmental processes that lead to nest destruction are often non-random: factors such as nest size, physical location, and network location are likely to be important in determining the likelihood that a nest is predated (e.g., Van Wilgenburg and Elgar 2007).

In polydomous ants, there is good evidence that sharing between nests occurs when one nest has excess resources and another nest has a resource deficit (Ellis et al. 2014). Consequently, polydomous colonies may have an advantage over monodomous colonies through being able to cover a larger area and absorb stochasticity in local environmental conditions (Holway and Case 2001), for example food production. However, we do not find support for this hypothesis. Instead, we find that polydomy is actually costly when food sources fluctuate in quality. The difference between the hypothesized result and the outcome of our model may be a consequence of small nests, common in polydomous colonies in our model, being more vulnerable to local reduction in food availability. Small nests may also be unable to grow quickly enough to fully exploit food sources that become very productive, an effect that has previously been found in models of polydomy (Cook et al. 2013). We demonstrate the logic of this hypothesized explanation using a simple model in Supplementary Appendix 7. Furthermore, there may be other situations not covered by our model, in which resource fluctuations result in higher frequencies of polydomy. For example, polydomous colonies may be better suited to surviving seasonal changes or spatial variation in resource availability than monodomous colonies, due to the ability of nests to share resources.

While our model shows increased foraging costs are associated with higher frequency of polydomy, we have not investigated the effect of altering resource sharing costs. Costs of resource sharing are likely to comprise a combination of time and energetic costs, and increased risk of predation and theft of transported resources while outside of the nest (Robinson 2014). We expect the relative costs of foraging and sharing are likely to be important to the profitability of polydomy in many different ant species. However, future work is necessary to determine the direction and effect of this interaction.

In this study, we focus on the adaptive benefits of either centralization of decentralization in ant colonies but have not considered the effects of genetic structure. Future work could adapt the model to include relatedness, which may provide important insights into the emergence of polydomous colonies. This is likely to be particularly important with respect to invasive supercolonies where population bottlenecks appear to be important (Giraud et al. 2002; Van Wilgenburg et al. 2010). We also do not look at how differences in the numbers of queens in each colony influence each of the hypotheses. The number of queens in polydomous colonies varies, with colonies of some species having many queens (polygynous) and colonies of other species having a single queen (monogynous) (Robinson 2014). This dichotomy is likely to have consequence for the risk spreading benefit of polydomy: if a nest in a monogynous colony containing the queen is predated, the colony will not survive if it is not able to rear a queen from existing brood. However, the influence of different numbers of queens per colony on the benefits of polydomy are likely to be less important for the other hypotheses because processes such as foraging are unlikely to be influenced by queen distribution. Future work could adapt our model to look at how each of the hypotheses we investigated are influenced by the number of queens in each colony.

Our research adds to a growing collection of studies that have used generative approaches—that is, process-based predictive models—to model biological networks (Seyfarth 1977; Cantor et al. 2015; Pinter-Wollman 2015; Ilany and Akçay 2016); the predictions of such models can then be tested empirically (Ilany and Akçay 2016). The advantage of using such models is that it is possible to test the logic of existing hypotheses and generate novel predictions about the way that different biological networks behave. The findings can then be used to inform the design of experiments. Our model highlights several key adaptive benefits to both centralization and decentralization in ecological systems, demonstrating how there may be multiple drivers of this trait and also helping to form testable hypotheses.

## FUNDING

This project was funded by NERC ACCE DTP (NE/L002450/1).

## Supplementary Material

arz138-suppl-Supplementary-Appendices

arz138-suppl-Supplementary-Video

arz138-suppl-Supplementary-Material

arz138-suppl-Supplementary-Dynamic-Model
