# Supplementary material for: The costs and benefits of decentralization and centralization of ant colonies
Source: Behav Ecol. 2019 Aug 14;30(6):1700–6. doi: 10.1093/beheco/arz138 (PMC6838651; doi:10.1093/beheco/arz138)
Supplement: arz138-suppl-Supplementary-Appendices [file arz138-suppl-supplementary-appendices.docx]

**Appendices**

**Appendix 1 - Model description**

**Purpose**

We compare the success of polydomy and monodomy across a range of environments with varying resource qualities and spatial distributions. We model the population dynamics of a community of ant nests - some of which have a polydomous colony organisation and others a monodomous colony organisation. The model is implemented in R Version 3.4.1.

**Environment and agents**

The environment is a continuous 2-dimensional area, containing a set number (Table A1.1) of food sources, each with fixed spatial coordinates. Each food source *i* produces food at constant rate *F_i_* throughout the season (parameter values in Table A1.2). This scenario is broadly based on our empirical systems where a 50x50m area contains many trees, each of which may act as an independent food source for ant nests.

The agents in the model represent nests, each of which belongs to one of several colonies. Nests contain ants, which are equivalent in the model to food (i.e. 1 food = 1 ant). Consequently, the model focuses on the behaviour of nests and does not model individual ants. Nests can either have a polydomous or monodomous colony organisation. If a nest’s colony organisation is polydomous then it can form outgoing connections to nests of the same colony, and to food sources. A nest whose colony organisation is monodomous can only form connections to food sources (see Figure 2 for graphical representation). Each nest takes food from food sources and nests - to which it has an outgoing connection - at a rate that is determined by a combination of biologically-motivated factors captured by Equations 3-5. Each nest is described by a nest identification number, a colony identification number, fixed spatial coordinates, a population size (*N_i_* for nest *i*), a colony organisation type (polydomous or monodomous), and a matrix of outgoing connections to food sources and nests.

**Table A1.1. Experimental method**

| **Description** | **Value** | **Units** |
| --- | --- | --- |
| Starting number of nests | 10 |  |
| Starting number of colonies | 10 |  |
| Number of food sources | 40 |  |
| Food source distribution | Random; Clustered |  |
| Length of season | 20 | arbitrary units |
| Number of seasons | 500 |  |
| Number of replicates per condition | 30 |  |
| Arena length/width | 50 | m |

**Processes**

**Overview and schedule**

After an initialisation stage, the model repeats over a set number of seasonal cycles. Within each season, there are six stages (2-7 in Figure A1.1) which are explained in detail below. We run the simulations for multiple independent replicates (see Appendix 8), each represented by a single complete run of the model for a fixed number of seasons (Table A1.1). For clarity of exposition, in the description of steps 2-6, the index of the season (i.e. the simulation “year”) is dropped.


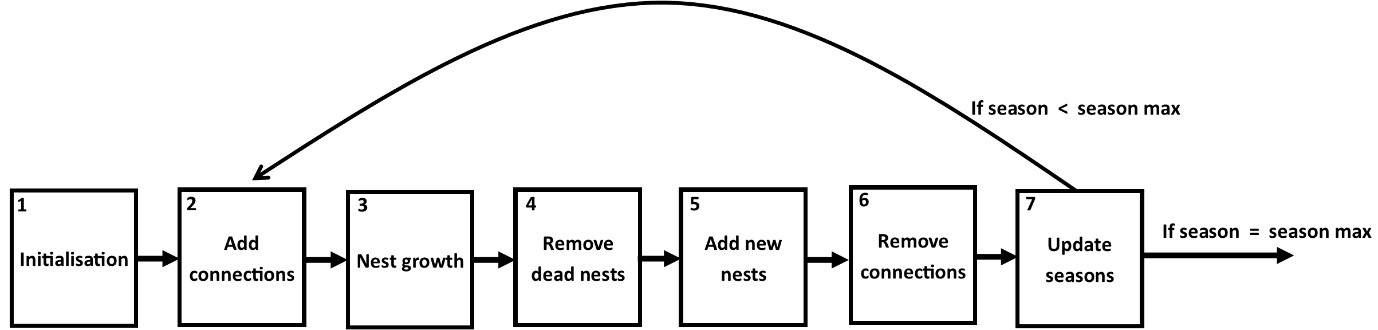


**Figure A1.1. Schedule of processes in model**

1. ***Initialisation***

During initialisation, the locations of food sources and nests are generated (Table A1.1). Each food source is allocated a unique identification number and is assigned location coordinates. The method for assigning the location of food sources depends on the condition and can be either random or clustered (details in Appendix 2). Nests are given unique nest and colony identification numbers, and their location coordinates are selected with a uniform random distribution over the arena. At initialisation all nests are assigned to belong to different colonies, with half of the initial nests having a polydomous colony organisation and the other half having a monodomous colony organisation (e.g. Figure 2). Initial nest sizes are selected from a uniform distribution between 0 and 100,000.

1. ***Add connections***

At the start of each season, each nest forms connections. These can connect the nest to food sources and - if the nest has a polydomous colony organisation - to other nests sharing the same colony identity. We assume that nests make more connections linearly depending on their population size. The number of connections *T_i_* that each nest is assigned in each season depends on the size of the nest *N_i_* (Table A1.3) and the number of ants needed to fully exploit a single food source in the constant food source condition ($\frac{F}{Q}$) (Equation 1). Finally, *T_i_* is rounded up to the nearest integer to give the number of connections that each nest is assigned. The number of connections that each nest adds is calculated by deducting the count of any connections that the nest has from previous seasons from *T_i_*.

$T_{i}= \lceil N_{i}\frac{Q}{F}\rceil$ **(1)**

The connections are then allocated. Nests are unable to detect how much food is available at a food source or nest until they are connected to it. However, nests are more likely to connect to food sources or other nests that are nearby. Thus, connections that would have a shorter length (*L_ij_* for a connection between nests *i* and *j*) are more likely to form than more distant connections. Formally, whenever nest *i* makes a new connection, the probability that the connection is to nest or resource *j* depends on the relative inverse squared distance of *j* from *i*, as quantified by Equation 2.

$P\left( connection from i to j \right)= \frac{{L_{ij}}^{-2}}{\sum_{n=1}^{\infty} {L_{in}}^{-2}}$ **(2)**

1. ***Nest growth calculations***

Once the network is established, changes in nest size are calculated for that season using a set of differential equations (Equations 3-7) modelling the continuous time dynamics of nests connected to a fixed set of nests and food sources throughout that season. These equations assume that nests take food from all food sources to which they are connected and - if they are polydomous - from nests to which they have an outgoing connection. The rate *R_ij_* that each nest *i* receives food from a connection to *j* depends on the number of ants A*_ij_* it commits to the connection (Equation 3), the amount of competition for that resource (Equation 4), the length of the connection (L*_ij_*), and the quantity of food that is available (see Equation 5).

We assume, for simplicity, that each nest commits an equal number of workers to each of its foraging trails. Therefore, number of ants (A*_ij_*) that a nest *i* commits to each of its connections *j* is calculated by dividing the total nest population size (*N_i_*) by the number of outgoing connections the nest has (*T_i_*) (Equation 3).

$A_{ij}= \frac{N_{i}}{T_{i}}$ **(3)**

Nests from different colonies compete for food from food sources, but do not compete for territory. We assume that the colony that has the largest number of ants foraging on a food source is the only colony able to take any food. However, for all colonies, the number of ants that take any food from the food source *j* is reduced proportionately to the sum of the effort by the other colonies that do not receive any food (Equation 4). We provide a sensitivity analysis of the inclusion of competition in Appendix 3.

$C_{ij}=\left\{ \begin{aligned} A_{ij}- \sum_{n=1}^{\infty} A_{nj}, \text{ if }\text{C}_{\text{ij}}\text{>0} \\ 0, \mathrm{otherwise} \end{aligned} \right.$ **(4)**

The rate at which food is taken by a foraging nest *i* from each food source or nest *j* to which it is connected is calculated by multiplying the number of individuals *A_ij_* from the nest committed to the connection, after costs of competition (*C_ij_*), by the quantity of food each individual can carry (*Q*). However, the rate at which the foraging nest receives food (*R_ij_*) is less than that taken from the food source or nest that it is foraging on, because of foraging costs. The foraging costs of a trail between nest *i* and nest or food source *j* are dependent on the number of foraging individuals (*A_ij_*) and the distance to the food source (*L_ij_*), which are multiplied by a constant connection cost (*E_ij_*), which is representative of energetic and trail maintenance costs, may differ for foraging trails and internest trails (Equation 5). The quantity of food it is possible to take from a food source is also limited by productivity (quantified by a rate *F_i_* for food source *i*), whereas, for other nests, the quantity is limited to a certain portion (*H*) of the nest population size (*N_i_*).

$R_{ij}=\left\{ \begin{aligned} QC_{ij}- A_{ij}L_{ij}E_{ij},\text{ if j is food source and }\text{R}_{\text{ij}}\text{ < }\text{F}_{\text{j}}\text{, or if j is nest and}\text{ }\text{R}_{\text{ij}}\text{ < }\text{N}_{\text{j}}\text{H} \\ F_{j}-A_{ij}L_{ij}E_{ij}, \text{otherwise, if j is a food source} \\ N_{j}H-A_{ij}L_{ij}E_{ij},\text{ }\text{ }\text{ otherwise, if j is a nest } \end{aligned} \right.$ **(5)**

Once *R_ij_* and *C_ij_* have been derived for all *i* and *j*, the rates of nest growth are calculated (Equation 6 and 7). The growth rate *G_i_* (Equation 6) of each nest *i* is determined by summing together all the food received from foraging, taking away the food lost to other nests and taking away a constant loss rate (*V*) (Table A1.2). Once this has been done, nest size is constrained by adjusting *G_i_* using a simple assumption of local logistic growth with *K* as the nest-level carrying capacity for each nest (Equation 7). This is incorporated to account for nest size constraints such as structural limitations (Robinson, 2014).

$G_{i}= \sum_{n=1}^{\infty} R_{in}-\sum_{n=1}^{\infty} R_{ni}+\sum_{n=1}^{\infty} A_{ni}L_{ni}E_{ni}- {VN}_{i}$ **(6)**

$\frac{dN_{i}}{dt}= G_{i}- G_{i}\frac{N_{i}}{K}$ **(7)**

The differential equations of nest growth are run for all the nests simultaneously over a fixed time interval for a given season (see Table A1.1; the units are arbitrary but may be considered as representing “weeks” during each seasonal “year”). The differential equations are solved using an ODE solver, built under the deSolve package (Karline Soetaert, 2010).

1. ***Remove dead nests***

At the end of the season, each nest which has a size below a threshold (*D*) is removed from future seasons. Additionally, each nest may ‘die’ stochastically, independent of size, with the probability of this occurring in each season determined by *P* (Table A1.2)*.* Nests that die through this process are also removed from future seasons.

1. ***Add new nests***

At the end of each season each nest can “parent” (produce through budding) a new nest, independently of all other nests, with a probability determined by dividing the nest’s current size by a constant (*U*), meaning that larger nests are more likely to create new nests (Equation 8). If a new nest is created, the location of the new nest depends on the colony organisation of the parent nest. If the parent nest is polydomous, the distance to the new nest from the parent nest is taken from a gamma distribution (see Parameterisation). The angle between the parent nest and the new nest is taken at random from a uniform distribution between 0º and 360º. The new nest location may be outside of the 50x50m environment. However, if the parent nest is monodomous, the new nest is given a random location in the arena. This method of nest foundation imitates independent foundation, whereby queens fly from the parent nest to found a new nest, which is commonly found in monodomous species (Pamilo & Rosengren, 1984). Once created, each new nest is given an initial size which is a fixed proportion *S* of the size of the parent nest. The size of the parent nest is reduced by the same amount. If the parent nest is polydomous then the new nest inherits the same colony identity and is also polydomous; otherwise it is given a new colony identity. However, every time a new nest is created there is a small probability (*M*) that the new nest will differ in colony organisation from the parent. If this occurs, then the new nest will have a new colony identity and its colony organisation will be different to that of its parent nest: if the parent was monodomous the new nest will form a new polydomous colony and if the parent was polydomous the new nest will form a new monodomous colony. Mutation of strategy in this way permits opportunities for either strategy to be re-established if it ever reaches extinction.

$P\left( i parents a nest \right)= \frac{N_{i}}{U}$ **(8)**

1. ***Remove connections***

To prepare for a new season, each connection can be either maintained or removed. Whether a connection is removed depends on the estimated profitability of the trail. The profitability of each connection from nest *i* to nest *j* (*R_ij_*) is calculated by Equation 5. We then take the inverse of *R_ij_* and multiply it by the nest size of *i* (*N_i_*), and a constant (*Z*) (Equation 9). *Z* is set to a value whereby more profitable connections are less likely to be removed than those with low profitability. Each connection that is entirely unprofitable (*R_ij_* < 0) is removed. If a connection is removed then it is not possible for the nest to remake the same connection in the next season. However, the connection may be made in any subsequent seasons.

$P\left( ij=1 \right)=min(Z\frac{R_{ij}}{N_{i}}, 1)$ **(9)**

1. ***Update seasons***

At the end of each season a season counter is updated. If the counter has not reached the final season (Table A1.1) the model will continue to Process 2, otherwise the replicate will be finished and will output the results.

**Table A1.2. Parameters, values and their units**

| **Letter** | **Description** | **Value** | **Units** |
| --- | --- | --- | --- |
| *F_i_* | Rate of productivity of food source *i* | Constant - 4000;  Fluctuating - selected from a uniform distribution between 0 and 8000 every season | resources t^-1^ |
| *K* | Carrying capacity | Low - 30,000;  High - 300,000 | ants |
| *Q* | Rate at which an ant can transport food to its nest along an outgoing connection | 0.1 | resources ant^-1^ t^-1^ |
| *H* | Rate of population size shared by polydomous nests | 0.01 | portion t^-1^ |
| *V* | Rate of colony loss | 0.05 | portion ^1^ t^-1^ |
| *U* | Constant to adjust probability of creating new nest | *K* x 20 | ants |
| *S* | Portion of a parent nest that is donated to a new nest | 0.1 |  |
| *D* | Minimum population threshold for nest survival to next season | 1000 | ants |
| *Z* | Constant to adjust probability of removing connection based on profitability | 0.001 |  |
| *E_ij_* | Connection cost for a connection from nest *i* to nest or food source *j* | Internest - 0.00001  Foraging (low) - 0.00001; Foraging (high) - 0.00005 | resources t^-1^ |
| *M* | Probability of strategy mutation | 0.1 |  |
| *P* | Probability of stochastic nest death | 0; 0.01 |  |

**Table A1.3. Output variables**

| **Letter** | **Description** | **Units** |
| --- | --- | --- |
| *N_i_* | Nest size of nest *i* | ants |
| *T_i_* | Number of outgoing connections from nest *i* |  |
| *L_ij_* | Length of connection from nest *i* to nest or food source *j* | cm |
| *A_ij_* | Number of ants committed to a connection from nest *i* to nest or food source *j* | ants |
| *C_ij_* | Number of ants committed to a connection after costs of competition from nest *i* to nest or food source *j* | ants |
| *R_ij_* | Resources received from a connection from nest *i* to nest or food source *j* | resources |

**Parameterisation**

The distance from a parent to a new nest for polydomous nests has been parameterised using six years of data on 10 colonies of polydomous wood ants (Ellis and Robinson (2015), DB *unpublished data*), which indicated a gamma distribution (fit described in Appendix 4).

**Appendix 2 – Food source placement**

Food sources are placed according to two different conditions: random and clustered. In the random condition food source locations are taken from a uniform distribution covering the whole arena (e.g. Figure A2.1A). In the clustered condition food sources are grouped into four clusters. Each cluster is given a centre, which is taken from a uniform distribution covering the whole arena, but with a minimum distance of 10m between them. Each food source is then given a location taken from a normal distribution with a mean that is equal to the cluster centre and a standard deviation of 2.5m (e.g. Figure A2.1B). If either of the x or y coordinates of any of the food sources is outside of the arena they are changed so that they are on the border of the arena.

**B**

**A**


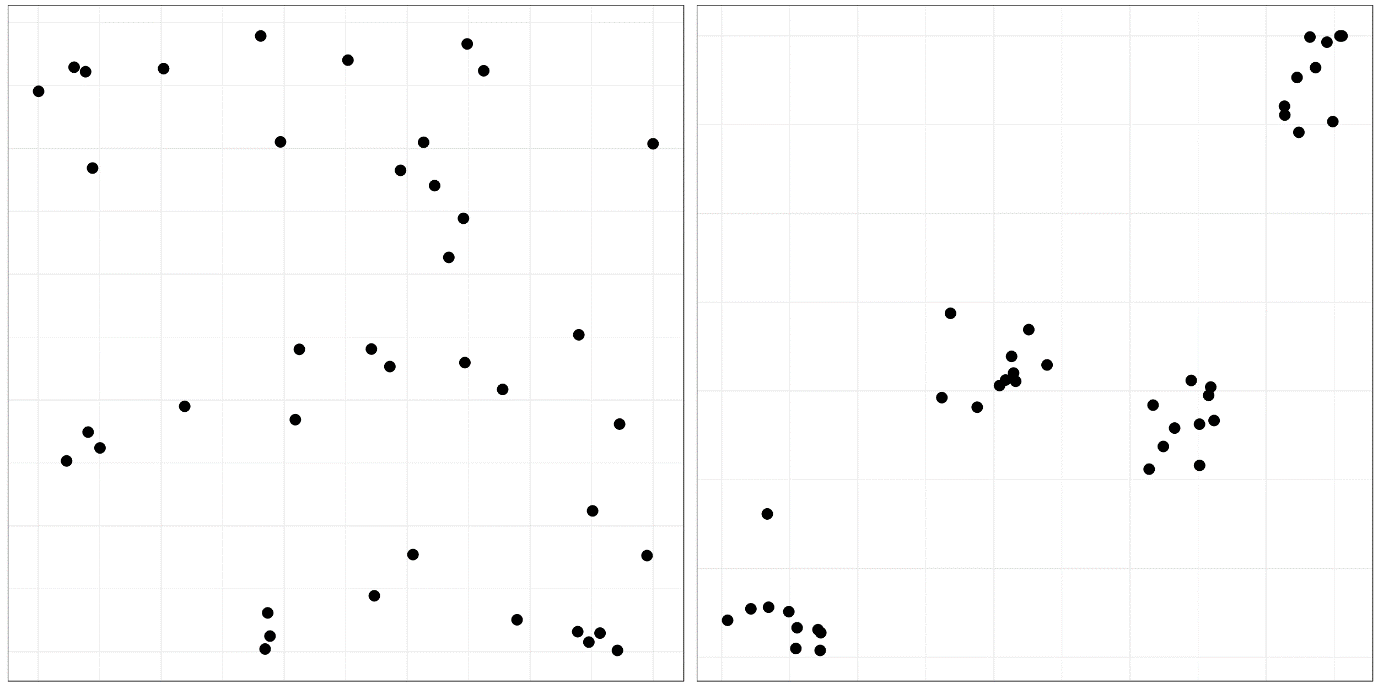


**Figure A2.1. Examples of the distribution of food sources for the random (A) and clustered (B) conditions. Each point is an individual food source.**

**Appendix 3 – Sensitivity analysis of competition**

To identify whether there is any effect of competition in the model on the relative success of polydomy and monodomy we repeat the method used in the main body of the paper, but remove direct competition (Equation 4) from the model. This was the only change we made to the model.

When competition is removed we find that in many of the conditions the population of nests grows very large. Consequently, competition is likely to be an important interaction to include in the model to prevent the population growing in an unrestricted way, as is likely to also be the case in many natural systems. Removing competition resulted in both a higher number of nests (Figure A3.1) and a higher total population (Figure A3.2). In addition, removal of competition appears to be favourable to monodomy (Figure A3.3/A3.4). This effect is unsurprising as competition is likely to be important to many of the advantages that polydomy appears to convey (e.g. resource monopolization). Consequently, the way that ant colonies compete is likely to play an important role in determining whether polydomy or monodomy is a better strategy.


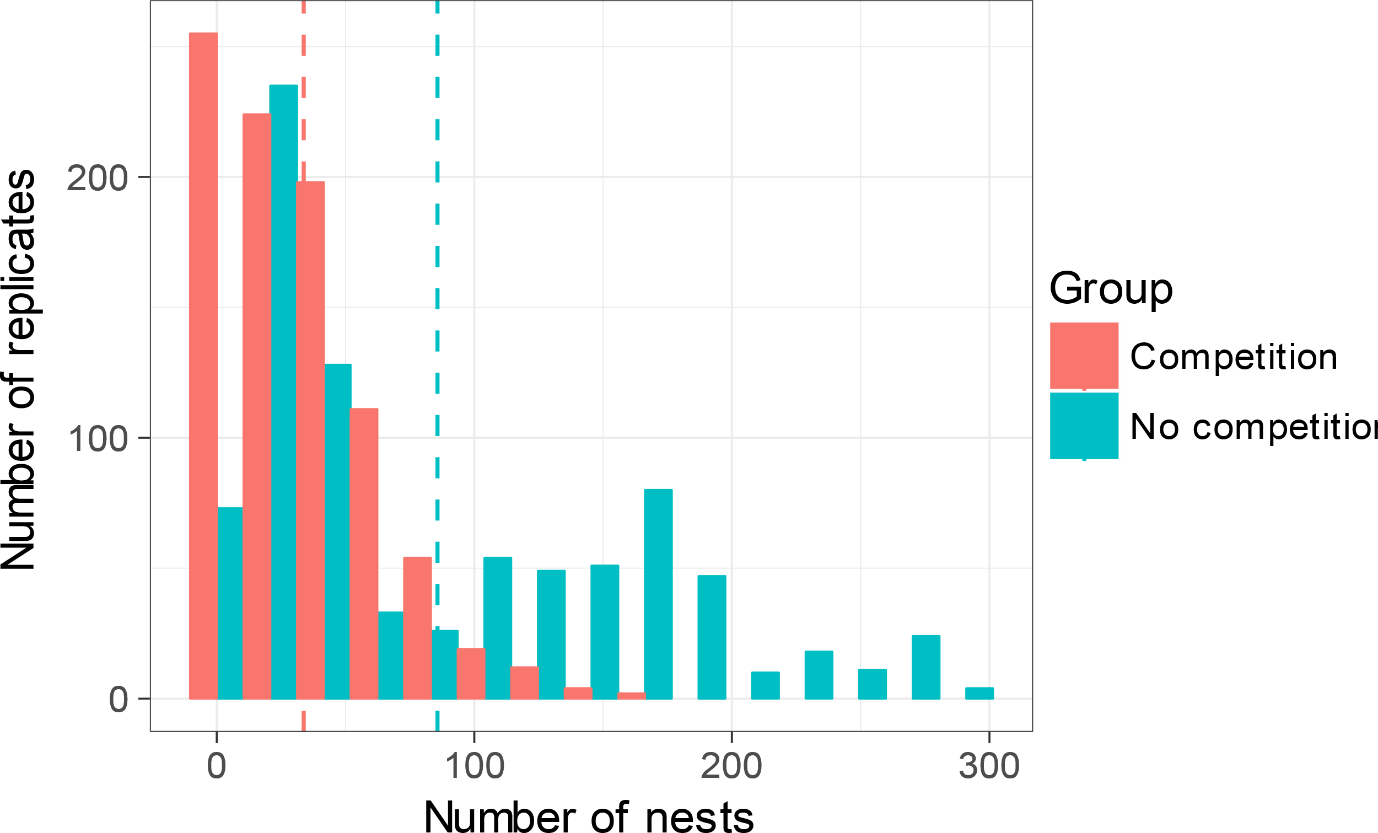


**Figure A3.1 Distribution of the number of nests in the final season of all replicates when competition is included (red) and when it is removed (blue). Vertical dashed lines represent median values.**

**
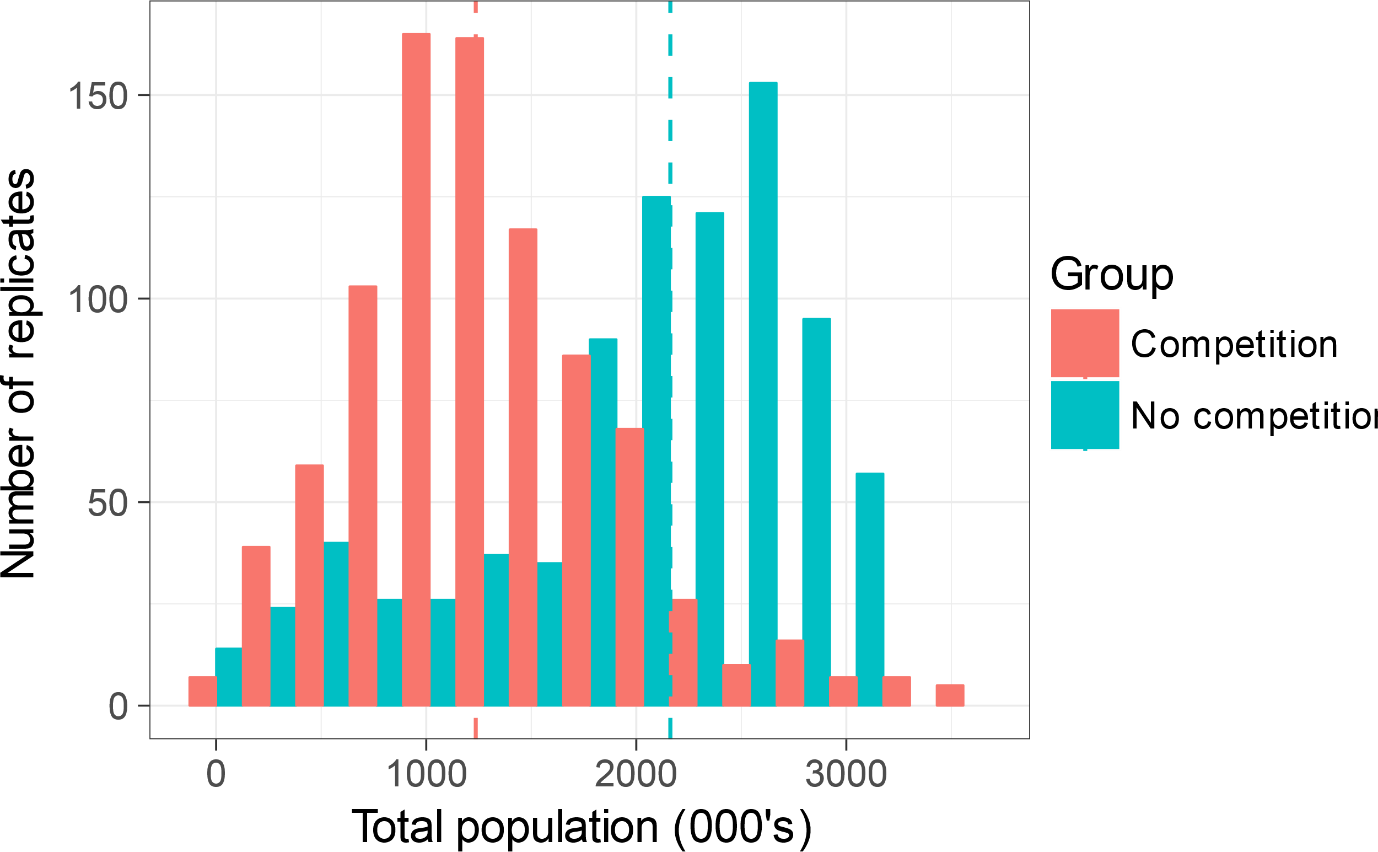
**

**Figure A3.2 Distribution of the total population size in the final season of all replicates when competition is included (red) and when it is removed (blue). Vertical dashed lines represent median values.**

**
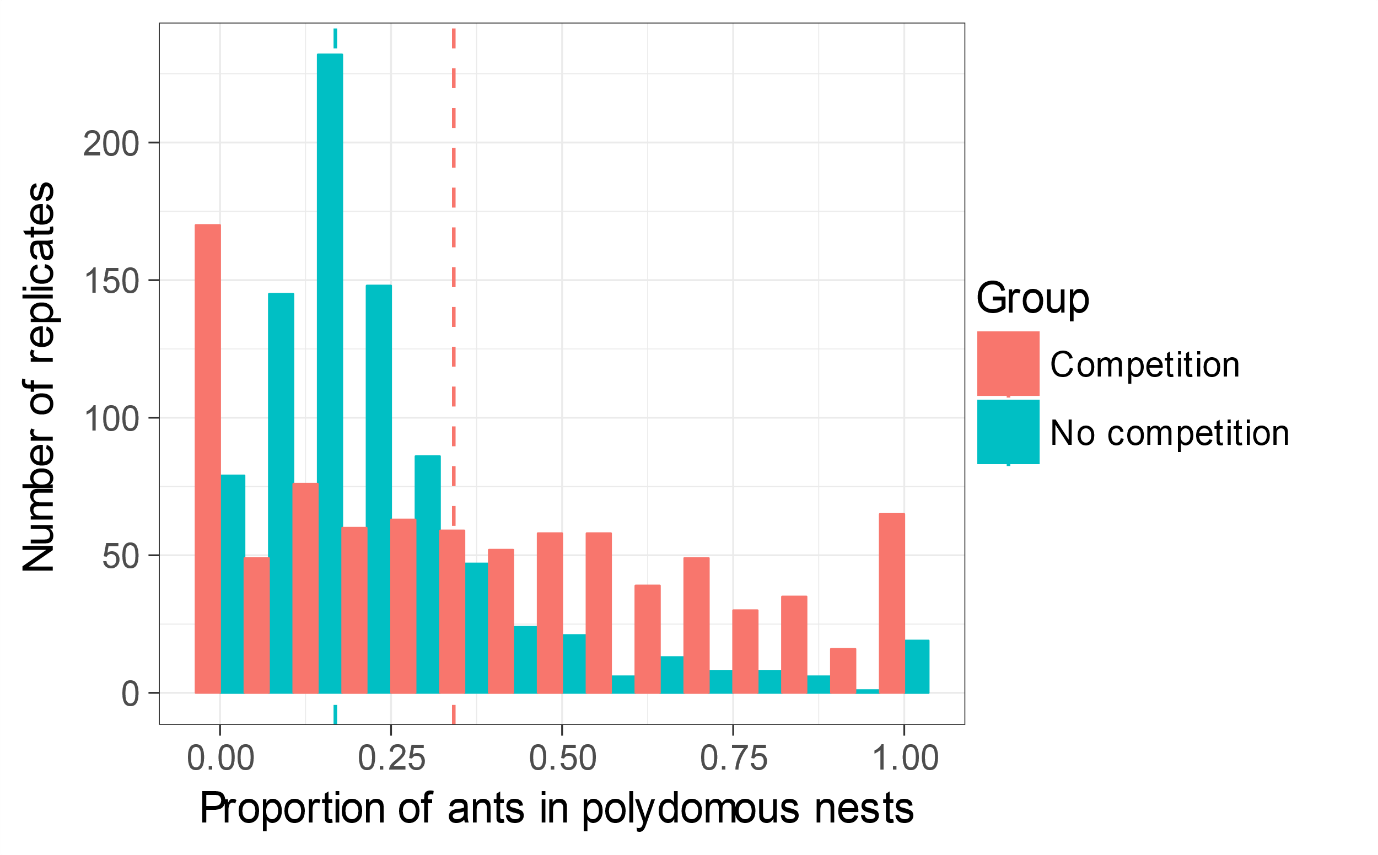
**

**Figure A3.3 Distribution of the proportion of the population in polydomous nests in the final season of all replicates when competition is included (red) and when it is removed (blue). Vertical dashed lines represent median values.**

**
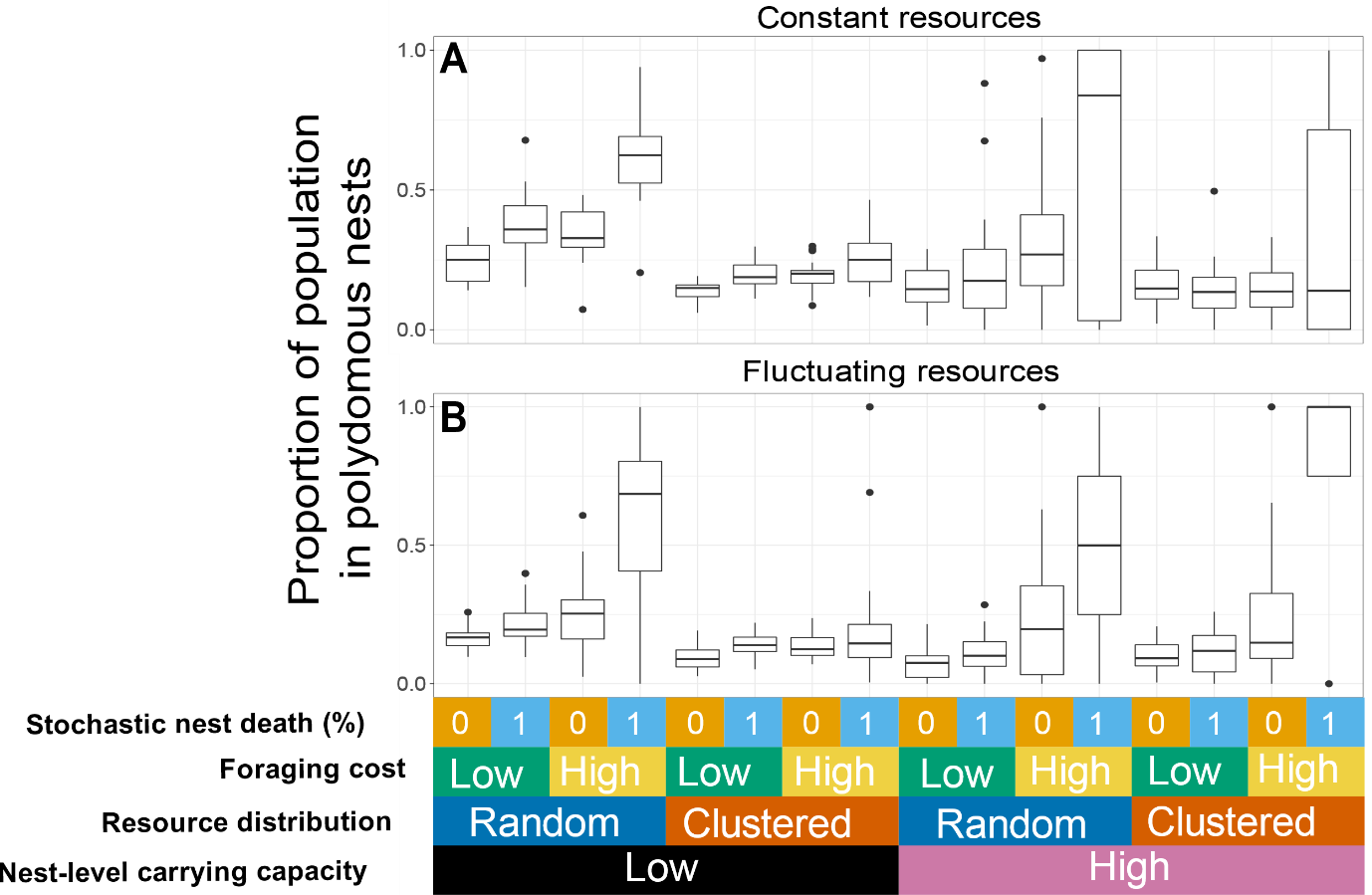
**

**Figure A3.4 The proportion of the population in polydomous nests at the end of 500 seasons in each condition when competition is removed. A shows conditions where food sources have a constant availability and B shows conditions where food sources fluctuate in availability. Middle lines represent median values, lower and upper hinges represent 25^th^ and 75^th^ percentiles respectively and whiskers reach to the lowest (lower whisker) or highest (higher whisker) value, with a maximum reach of 1.5 x IQR from the hinge. Values outside of this range are plotted as outliers.**

**Appendix 4. Fit of gamma distribution to founding data**

Over the last seven years data on 10 polydomous colonies of northern hairy wood ants (*Formica lugubris*) have been collected (Ellis et al. 2015; DB *unpublished data*). We identified cases where new nests had been founded and the identity of the parent nest was clear. We measured the distance from the parent nest to the new nest and fit a gamma distribution to these distances. Diagnostic plots of the model fit indicated a good fit (Figure A4.1).

**
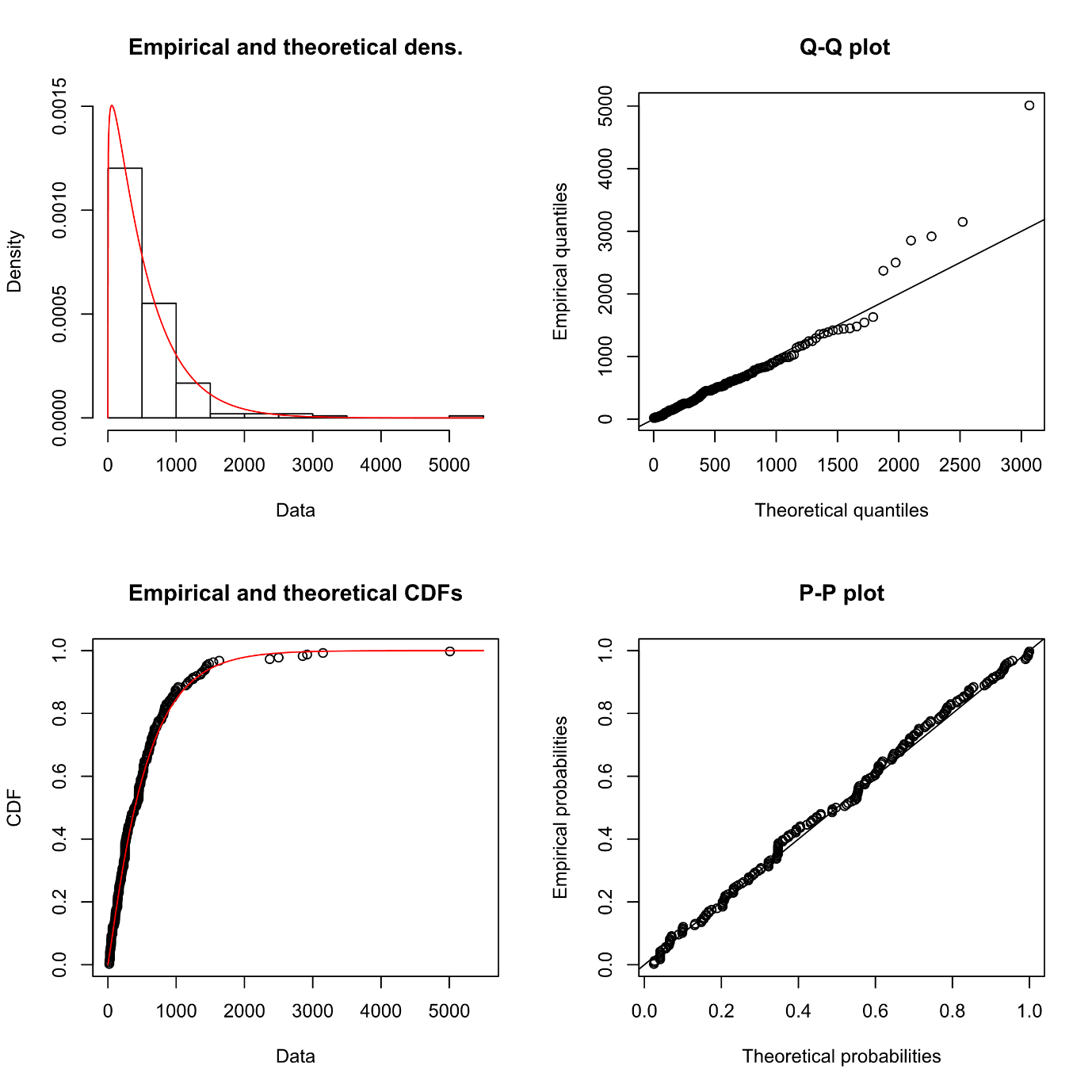
**

**Figure A4.1. Fit of the gamma model to the founding distance data.**

**Appendix 5 – Model selection**

We fit a binomial general linear model with a logit link to the data and selected a final model by first fitting all single factors and two-way interactions between factors in the data and then removing factors in step-wise fashion, only keeping any changes that resulted in a model with a lower AIC. Each of the models tested and their AICs are as follows:

**Table A5.1 AIC for different models used in the model selection. Predictors are coded as follows: S = food stochasticity, K = nest-level carrying capacity, F = foraging trail cost, D = stochastic nest death and R = food distribution. Letters separated by a colon indicate an interaction between the two factors. The final model is indicated in bold text.**

| **Model** | **Predictors** | **AIC** |
| --- | --- | --- |
| 1 | Single: S, K, F, D, R  Interactions: S:K, S:F, S:D, S:R, K:F, K:D, K:R, F:D, F:R, D:R | 945.95 |
| 2 | Single: S, K, F, D, R  Interactions: S:K, S:F, S:D, S:R, K:F, K:D, K:R, F:D, D:R | 944.44 |
| 3 | Single: S, K, F, D, R  Interactions: S:K, S:F, S:R, K:F, K:D, K:R, F:D, D:R | 942.42 |
| 4 | Single: S, K, F, D, R  Interactions S:K, S:F, S:R, K:F, K:D, K:R, F:D | 939.54 |
| 5 | Single: S, K, F, D, R  Interactions: S:K, S:F, S:R, K:F, K:D, K:R | 938.2 |
| 6 | Single: S, K, F, D, R  Interactions: S:K, S:F, S:R, K:D, K:R | 935.32 |
| **7** | **Single: S, K, F, D, R**  **Interactions: S:K, S:R, K:D, K:R** | **934.67** |
| 8 | Single: S, K, F, D, R  Interactions: S:K, S:F, K:D, K:R | 952.46 |

**Appendix 6 – Population extinction in different conditions of the model**


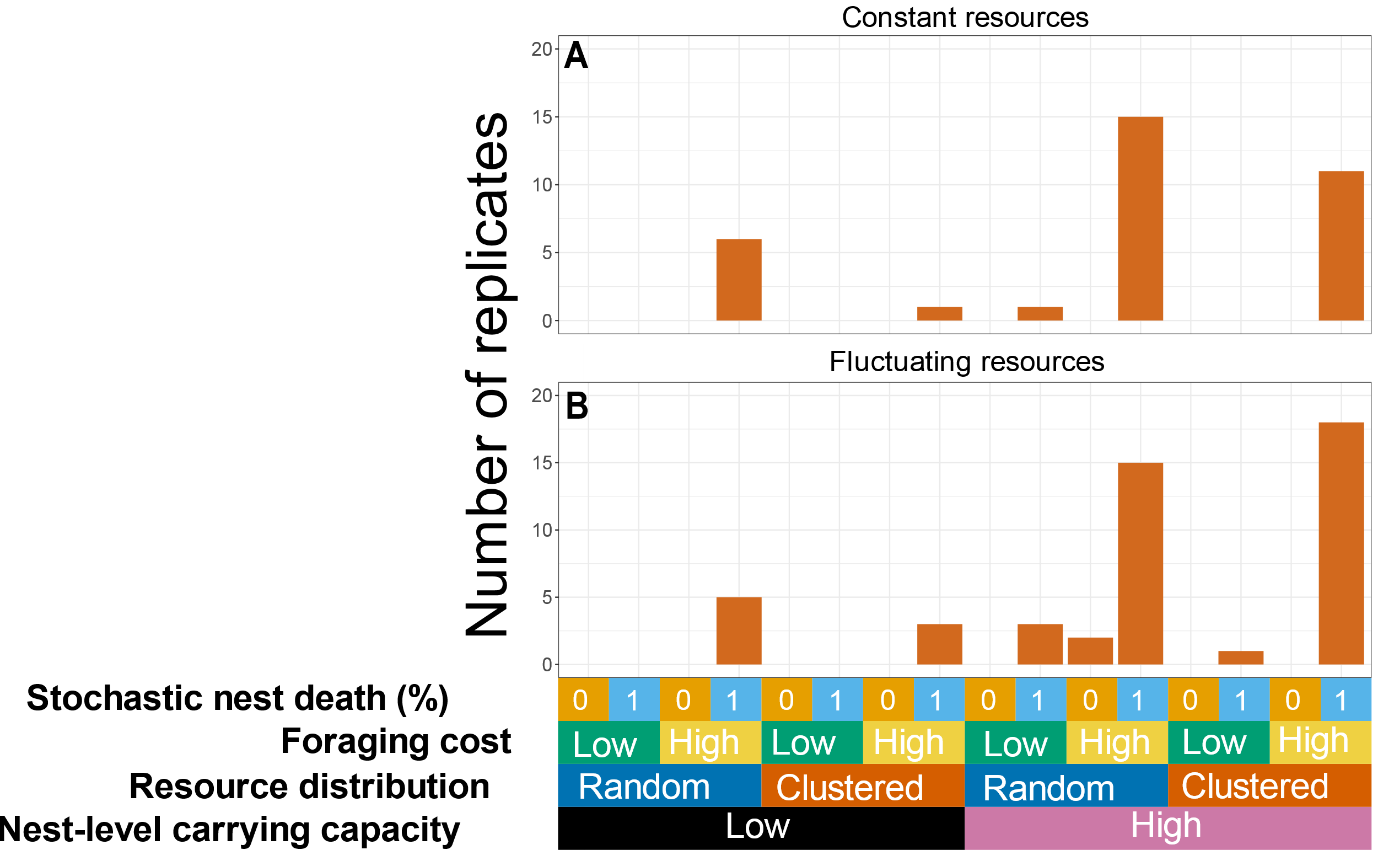


**Figure A6.1. The number of replicates, out of 30, in which the population did not survive until the final season** **in each condition when food is either constant (A) or fluctuating (B) in availability.**

**Appendix 7 – Growth of different sized nests in response to different quantities of food**

Here we present a simple mathematical model demonstrating the growth of individual nests when presented with food sources of different size. The model uses the same equations as in the main model. However, in each simulation there is only a single nest alive that is placed 5m from two food sources that each have the same size, which is determined by the condition (Table A7.1). Consequently, there is no competition or internest sharing. For simplicity, we also remove the ability of nests to parent new nests.

**Table A7.1 Parameters, their values and units used in model for Appendix 7.**

| **Letter** | **Description** | **Value** | **Units** |
| --- | --- | --- | --- |
| *F_i_* | Rate of productivity of food source *i* | 0; 8,000 | Resources t^-1^ |
| *K* | Carrying capacity | 300,000 | Ants |
| *Q* | Rate at which an ant can transport food to its nest along an outgoing connection | 0.1 | Resources ant^-1^ t^-1^ |
| *V* | Rate of colony loss | 0.05 | Portion ^1^ t^-1^ |
| *E* | Connection cost | 0.00005 | Resources t^-1^ |

We test the growth of two different sized nests (small = 5,000, large = 100,000) under two different conditions of *F_i_*, which represent situations where food sources fluctuate to be either unproductive or very productive in the ‘fluctuating’ condition in the main model. We run the model for a length of 200 arbitrary units of time.

The results from this basic model are presented in Figure A6.1 and demonstrate that smaller nests grow more slowly when presented with very productive food sources, but also become extinct faster when presented with unproductive food sources.

**
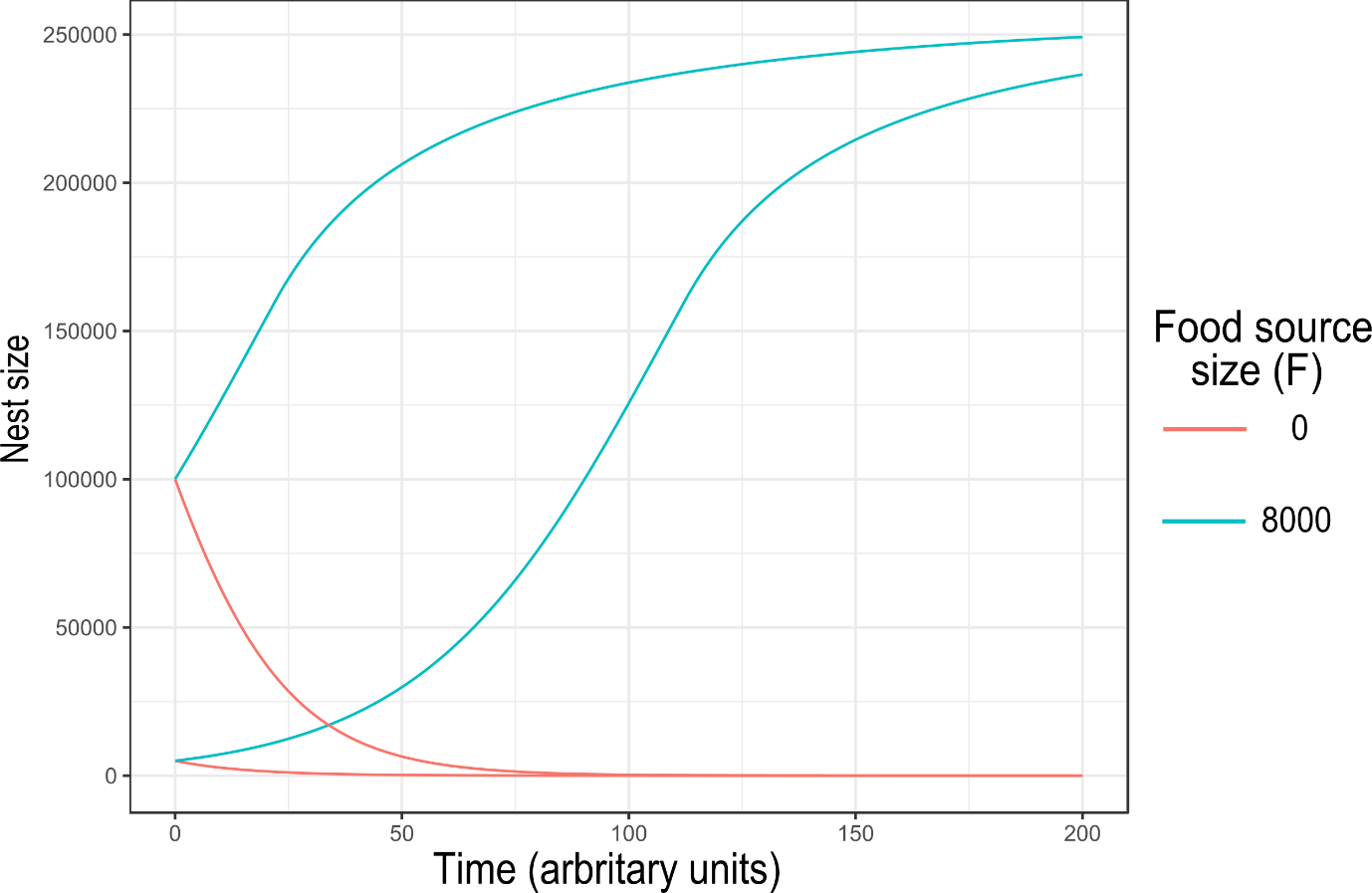
Figure A7.1. Growth of nests of different population size when presented with different food sources.**

**Appendix 8 – Response of cumulative mean of frequency of polydomy in response to number of replicates**

Here we present a plot demonstrating how cumulative mean of the frequency of polydomy changes as more replicates are added to each condition. In most of the conditions, there is very little change in the mean as more replicates are added once there are 30 replicates. Consequently, 30 replicates is sufficient to give reliable results.


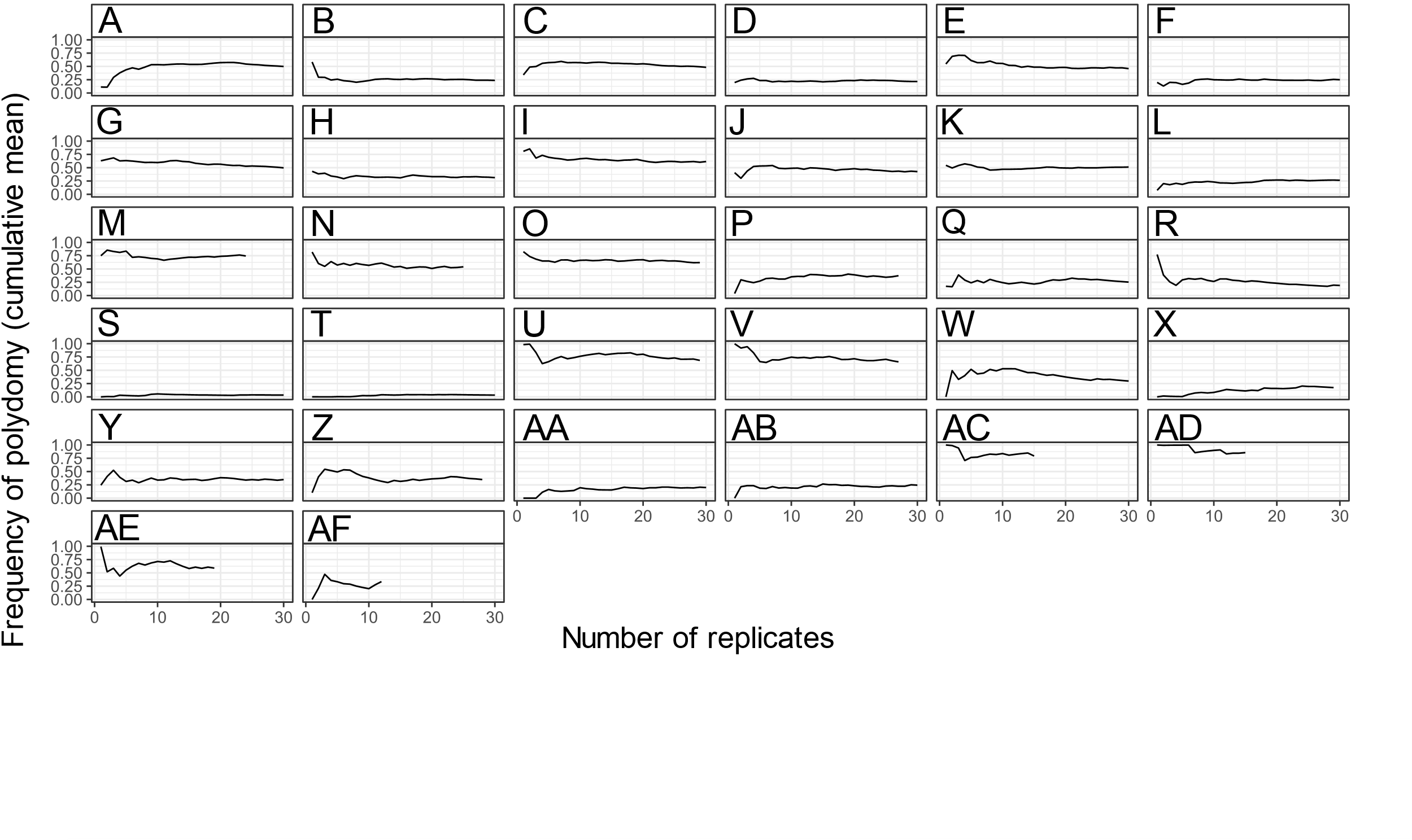


**Figure A8.1 Change in the cumulative mean of frequency of polydomy as more replicates are added in each condition. Conditions are given in Table A8.1. Some conditions have fewer than 30 records as populations did not always survive to the final timestep (see Appendix 6).**

| Letter | Nest-level carrying capacity | Foraging cost | Stochastic nest death | Distribution of resources | Food source productivity |
| --- | --- | --- | --- | --- | --- |
| A | Low | Low | None | Clustered | Constant |
| B | Low | Low | None | Clustered | Fluctuating |
| C | Low | Low | None | Random | Constant |
| D | Low | Low | None | Random | Fluctuating |
| E | Low | Low | 1% | Clustered | Constant |
| F | Low | Low | 1% | Clustered | Fluctuating |
| G | Low | Low | 1% | Random | Constant |
| H | Low | Low | 1% | Random | Fluctuating |
| I | Low | High | None | Clustered | Constant |
| J | Low | High | None | Clustered | Fluctuating |
| K | Low | High | None | Random | Constant |
| L | Low | High | None | Random | Fluctuating |
| M | Low | High | 1% | Clustered | Constant |
| N | Low | High | 1% | Clustered | Fluctuating |
| O | Low | High | 1% | Random | Constant |
| P | Low | High | 1% | Random | Fluctuating |
| Q | High | Low | None | Clustered | Constant |
| R | High | Low | None | Clustered | Fluctuating |
| S | High | Low | None | Random | Constant |
| T | High | Low | None | Random | Fluctuating |
| U | High | Low | 1% | Clustered | Constant |
| V | High | Low | 1% | Clustered | Fluctuating |
| W | High | Low | 1% | Random | Constant |
| X | High | Low | 1% | Random | Fluctuating |
| Y | High | High | None | Clustered | Constant |
| Z | High | High | None | Clustered | Fluctuating |
| AA | High | High | None | Random | Constant |
| AB | High | High | None | Random | Fluctuating |
| AC | High | High | 1% | Clustered | Constant |
| AD | High | High | 1% | Clustered | Fluctuating |
| AE | High | High | 1% | Random | Constant |
| AF | High | High | 1% | Random | Fluctuating |

**Table A8.1. Conditions for Figure A8.1**

**Appendix 9 – Sensitivity analyses for parameters**

Here we present sensitivity analyses for parameters in the model. For each parameter we ran 30 replicates where we decreased the parameter by 10% and 30 replicates where we increased the parameter by 10%. All parameters other than the focal parameter were not changed. We performed a linear model on the results with each of the results to check whether any of the parameters heavily influenced the proportion of population in polydomous nests. The p-values from the linear model were then corrected for multiple comparisons using the *Bonferonni-Holm* method. The sensitivity analyses demonstrate that none of the parameters heavily influence the frequency of polydomy in the model (Figure A9.1/Table A9.1) .


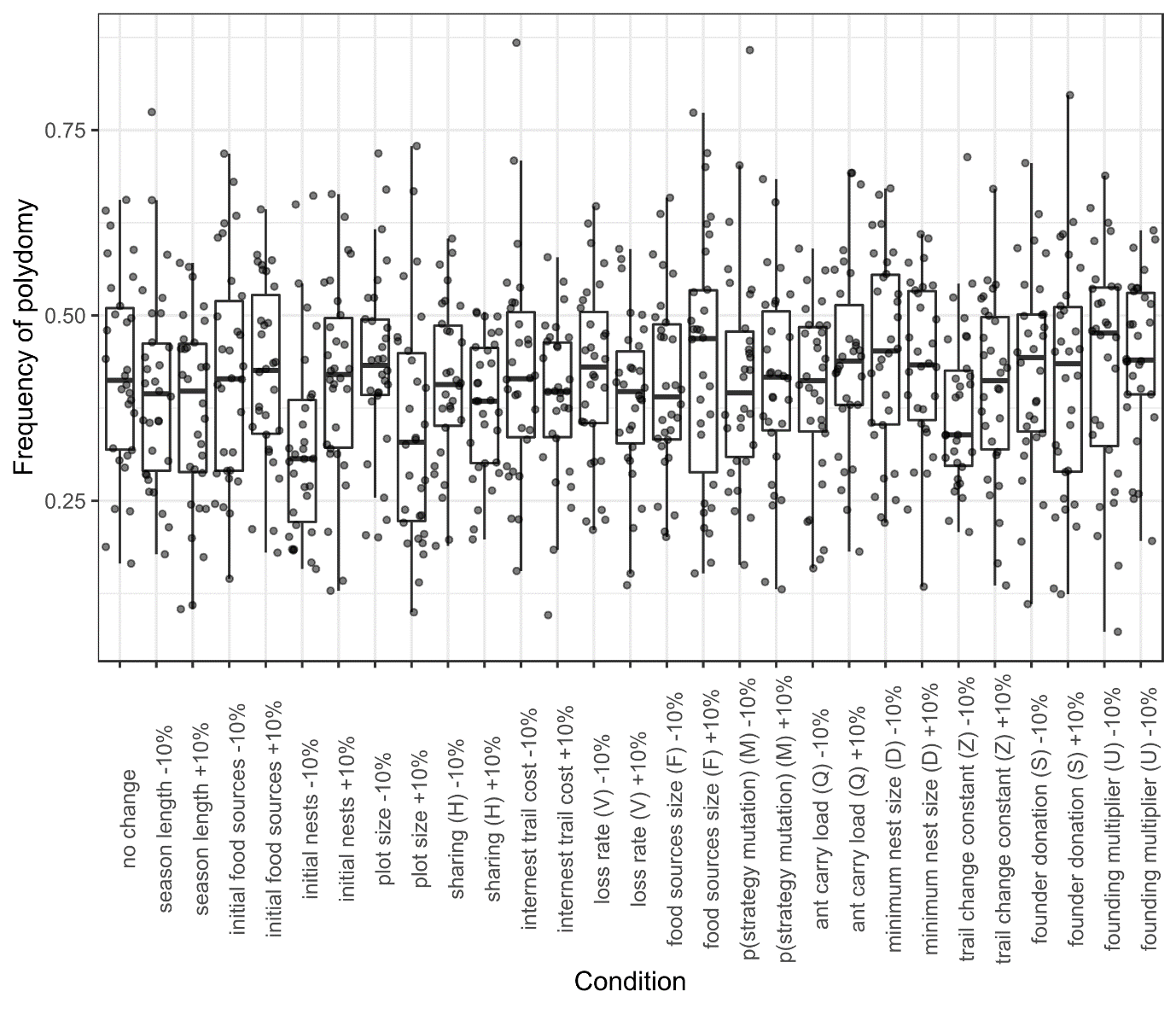


**Figure A9.1. Results of sensitivity analyses the model. Letters for parameters are given where relevant. Middle lines represent median values, lower and upper hinges represent 25^th^ and 75^th^ percentiles respectively and whiskers reach to the lowest (lower whisker) or highest (higher whisker) value, with a maximum reach of 1.5 x IQR from the hinge. All values are plotted as jittered points.**

**Table A9.1. Results from the linear model of sensitivity analyses. The frequency of polydomy in each condition is compared against the frequency of polydomy in the control condition.**

| **Condition** | **Estimate** | **Standard error** | **T** | **Adjusted p-value** |
| --- | --- | --- | --- | --- |
| season length -10% | -0.02 | 0.03 | -0.70 | 0.98 |
| season length +10% | -0.05 | 0.03 | -1.55 | 0.87 |
| initial food sources -10% | 0.00 | 0.03 | 0.00 | 1.00 |
| initial food sources +10% | 0.00 | 0.03 | -0.03 | 1.00 |
| initial nests -10% | -0.09 | 0.03 | -2.80 | 0.08 |
| initial nests +10% | -0.01 | 0.03 | -0.23 | 0.98 |
| plot size -10% | 0.01 | 0.03 | 0.37 | 0.98 |
| plot size +10% | -0.08 | 0.03 | -2.29 | 0.22 |
| sharing (H) -10% | -0.01 | 0.03 | -0.40 | 0.98 |
| sharing (H) +10% | -0.05 | 0.03 | -1.33 | 0.88 |
| internest trail cost -10% | 0.00 | 0.03 | 0.05 | 1.00 |
| internest trail cost +10% | -0.03 | 0.03 | -1.01 | 0.98 |
| loss rate (V) -10% | 0.00 | 0.03 | 0.01 | 1.00 |
| loss rate (V) +10% | -0.04 | 0.03 | -1.04 | 0.98 |
| food sources size (F) -10% | -0.01 | 0.03 | -0.41 | 0.98 |
| food sources size (F) +10% | 0.01 | 0.03 | 0.42 | 0.98 |
| p(strategy mutation) (M) -10% | -0.01 | 0.03 | -0.20 | 0.98 |
| p(strategy mutation) (M) +10% | -0.01 | 0.03 | -0.41 | 0.98 |
| ant carry load (Q) -10% | -0.03 | 0.03 | -0.77 | 0.98 |
| ant carry load (Q) +10% | 0.02 | 0.03 | 0.60 | 0.98 |
| minimum nest size (D) -10% | 0.03 | 0.03 | 0.83 | 0.98 |
| minimum nest size (D) +10% | 0.01 | 0.03 | 0.32 | 0.98 |
| trail change constant (Z) -10% | -0.05 | 0.03 | -1.44 | 0.87 |
| trail change constant (Z) +10% | -0.02 | 0.03 | -0.59 | 0.98 |
| founder donation (S) -10% | 0.01 | 0.03 | 0.22 | 0.98 |
| founder donation (S) +10% | -0.01 | 0.03 | -0.31 | 0.98 |
| founding multiplier (U) -10% | 0.01 | 0.03 | 0.30 | 0.98 |
| founding multiplier (U) -10% | 0.02 | 0.03 | 0.65 | 0.98 |
